# Tie-Line Analysis Reveals Interactions Driving Heteromolecular Condensate Formation

**DOI:** 10.1103/PhysRevX.12.041038

**Published:** 2022-12-30

**Authors:** Daoyuan Qian, Timothy J. Welsh, Nadia A. Erkamp, Seema Qamar, Jonathon Nixon-Abell, Georg Krainer, Peter St. George-Hyslop, Thomas C. T. Michaels, Tuomas P. J. Knowles

**Affiliations:** 1 Centre for Misfolding Diseases, Yusuf Hamied Department of Chemistry, University of Cambridge, Lensfield Road, Cambridge, CB2 1EW, United Kingdom; 2 Cambridge Institute for Medical Research, Department of Clinical Neurosciences, Clinical School, University of Cambridge, Cambridge, CB2 0XY, United Kingdom; 3 Department of Medicine (Division of Neurology), Temerty Faculty of Medicine, University Health Network, University of Toronto, Toronto, Ontario M5T 0S8, Canada; 4 Department of Neurology, Columbia University, 710 West 168th Street, New York, New York 10032, USA; 5 Department of Physics and Astronomy, Institute for the Physics of Living Systems, University College London, London WC1E 6BT, United Kingdom; 6 Cavendish Laboratory, Department of Physics, University of Cambridge, J. J. Thomson Avenue, Cambridge, CB3 0HE, United Kingdom

**Keywords:** Biological Physics, Interdisciplinary Physics, Soft Matter

## Abstract

Phase separation of biomolecules gives rise to membraneless organelles that contribute to the spatiotemporal organization of the cell. In most cases, such biomolecular condensates contain multiple components, but the manner in which interactions between components control the stability of condensates have remained challenging to elucidate. Here, we develop an approach to determine tie-line gradients in ternary biomolecular phase-separation systems based on measurements of the dilute phase concentration of only one component. We show that the sign of the tie-line gradient is related to the cross-interaction energy between the polymers in the system and discriminates between associative and segregative phase separation. Using this approach, we study the interaction between protein fused in sarcoma (FUS) and polyethylene glycol (PEG) polymer chains and measure positive tie-line gradients. Our results show that PEG drives phase separation through an associative interaction with FUS, other than through acting as an inert crowder. We further study the interaction between poly(A) ribonucleic acid (RNA) (3.0 ± 0.5 kDa [kilodalton]) and the protein G3BP1, and using the tie-line gradient as a reporter for the stoichiometry of polymers in the condensate, we determine a G3BP1-to-poly(A) RNA molar ratio of 1 : 4 in the dense phase. Our framework for measuring tie-line gradients opens up a route for the characterization of interaction types and compositions in ternary phase-separation systems.

## I Introduction

Biomolecular condensates play important roles in cells, both in healthy physiological function and disease development [1–7]. These condensates form through phase separation and in many cases involve interactions between different biomolecules such as protein and ribonucleic acid (RNA) [8,9]. This phenomenon is often studied by generating phase diagrams that show under what conditions condensates form [10]. However, experimentally measured phase diagrams only partially cover the phase space and contain limited information on whether solutes colocalize in the condensate or prefer to be in separate phases, and this information is instead contained in tie lines. A tie line is defined in the following manner: Take a ternary phase-separating system with two solutes—a protein and an agent—and induce phase separation by mixing a suitable amount of each. The dilute and dense phases contain a given concentration of the protein and the agent, and we can plot the two phases as two points on the phase diagram with concentrations as their coordinates. The tie line is the line connecting these two points, as such a line indicating if the solutes prefer to be in the same or different phases, giving a positive or negative tie-line gradient, respectively. Tie lines give rise to variable phase compositions depending on total solute concentrations [11,12], and as a result, they can potentially make a phase-separating system less prone to density fluctuations [12,13] or affect reaction kinetics [14]. Tie lines have been investigated computationally before [12,15–17], while they remain elusive in experimental settings since their determination formally requires measurement of all solute concentrations in both phases [18].

In this paper, we aim to connect experimental measurement and theoretical analysis of tie-line gradients via simple approaches. Theoretically, using the Flory-Huggins model we find that tie-line directions directly relate to the cross-interaction between solutes. In the case of a positive gradient, we can further relate the gradient to the stoichiometry of solutes in the dense phase. Experimentally, we demonstrate tie-line gradients can be determined by measuring the concentration of only one solute in the dilute phase. We do not require measurement of the other solute or dense phase concentrations, both enabling investigations with solutes that cannot be easily labeled or measured and ensuring minimal artificial effect from florescent tags [19]. Here we study three systems. The first and second involve the protein fused in sarcoma (FUS). FUS is a 75-kDa nucleic-acid-binding protein known to be key for controlling gene expression across cell types [20,21] and closely related to the neurode-generative disease amyotrophic lateral sclerosis [22]. FUS is also known to undergo phase separation in response to many stimuli including salt [23], RNA [24], and polyethylene glycol (PEG), where PEG was previously assumed to be an inert crowder [22,25]. We investigate phase separation of FUS with either PEG20k (20 kDa) or PEG10k (10 kDa). In both systems, we find FUS and PEG colocalize in condensates, meaning PEG does not act as a crowder. Our third system involves the protein G3BP1, a 60-kDa GTP-ase activating protein which has RNA-binding properties. G3BP1 is known to be the core protein responsible for nucleating the formation of stress granules [8,26], a type of membraneless organelle responsible for controlling stress response in cells [1,27], and G3BP1 is also associated with cancer development [28,29]. As part of its role in stress granule formation, G3BP1 forms favorable interactions with RNA to induce formation of condensates [1]. We study the interaction between G3BP1 and single-stranded poly(A) RNA (3.0 ± 0.5 kDa) and find that G3BP1 and the poly (A) colocalize in the condensate with a molar ratio of 1 : 4. Our method can be widely applied to characterize interactions and compositions of condensates in other phase-separating systems too.

## II Solute Interaction Directly Relates to Tie-Line Gradient

Here we use the Flory-Huggins model to investigate tie lines. The Flory-Huggins free-energy density *f* of mixing a solvent with volume fraction *ϕ*_0_, and two different polymer solutes with lengths *N*_1_, *N*_2_ and volume fractions *ϕ*_1_, *ϕ*_2_, respectively, is [30–33]

(1) $$\begin{array}{c} \frac{f}{k_{B}T}=\phi_{0}\text{ln}\phi_{0}+\frac{\phi_{1}}{N_{1}}\text{ln}\phi_{1}+\frac{\phi_{2}}{N_{2}}\text{ln}\phi_{2}\\ +\chi_{01}\phi_{0}\phi_{1}+\chi_{02}\phi_{0}\phi_{2}+\chi_{12}\phi_{1}\phi_{2} \end{array}$$

with *k*_*B*_*T* the unit thermal energy, *χ*_*μν*_ the effective interaction between components *μ* and *ν* (*μ, ν* = 1, 2), and the solvent volume fraction is constrained by the condition *ϕ*_0_ =1 − *ϕ*_1_ − *ϕ*_2_. The first three terms in Eq. (1) are entropic contributions that favor mixing, while the remaining terms are interactions that can favor either mixing or phase separation, depending on values of *χ*_*μν*_.

Taken together, the free-energy-density landscape *f ϕ*_1_; *ϕ*_2_ can be used to deduce phase-separation propensity at a particular combination of solute concentrations (*ϕ*_1_; *ϕ*_2_): If the free-energy density is concave in any direction, the system is susceptible to small thermal fluctuations and will phase separate following Cahn-Hilliard dynamics [34,35]. This region of the phase diagram is the spinodal; on the other hand, a locally convex free-energy density traps the system in a “valley” and the mixture is locally stable. Outside the spinodal region, phase separation is still possible if total free energy of the system can be reduced by having compartments with distinct solute compositions, resulting in a global free-energy minimization problem with constrained total volume and solute mass [36,37]. The region of phase space where such a total free-energy reduction via compartmentalization is possible is the binodal region, and it completely encompasses the spinodal region. In the space within the binodal but outside the spinodal, the phase-separation dynamics is instead governed by nucleation of the new phase with an initial free-energy barrier.

**Fig. 1:**
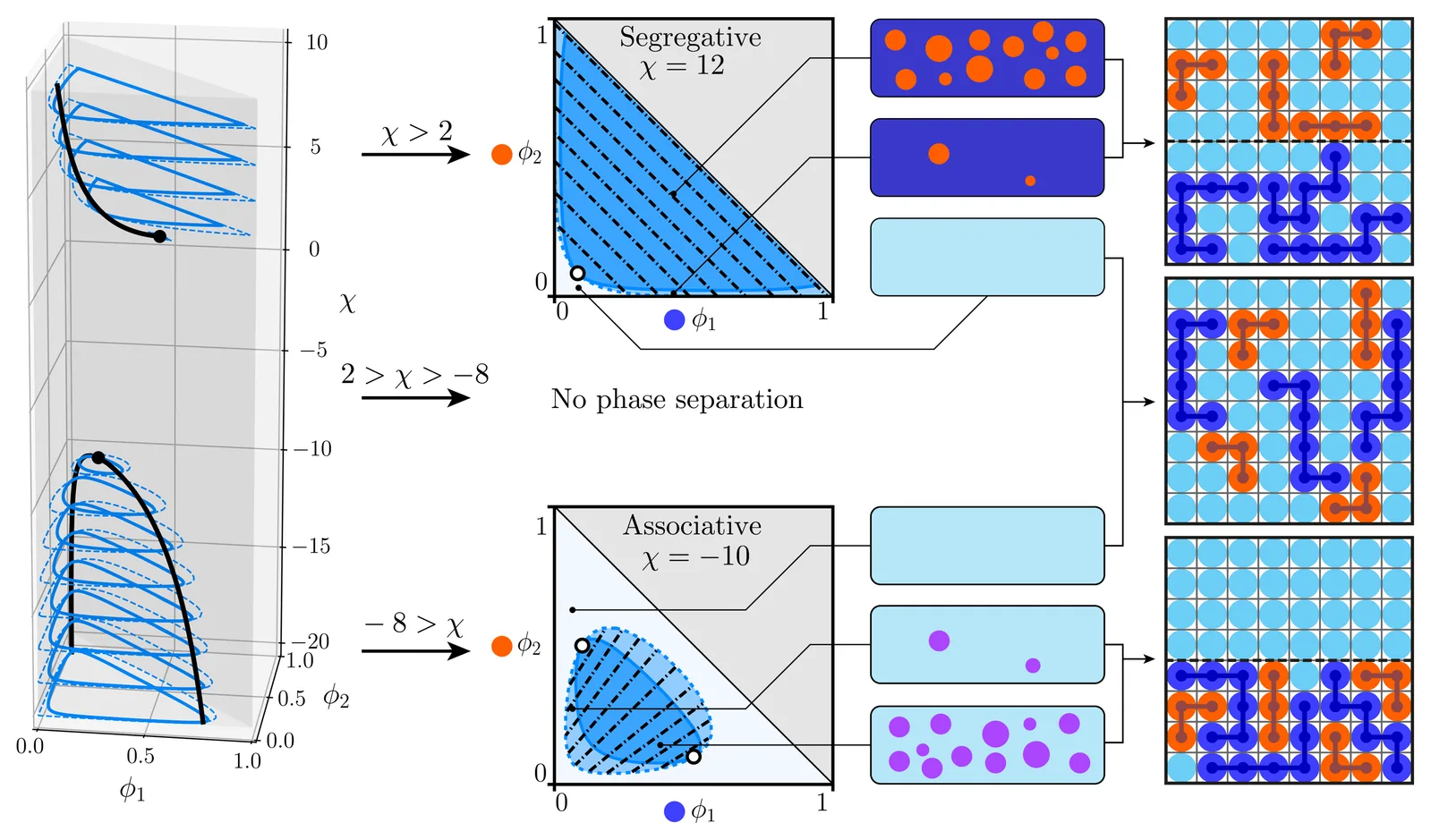
Phase-space structure of a ternary system. Left column: critical points (black dots and black lines), spinodal boundaries (blue solid lines), and binodal boundaries (blue dashed lines) in the (*ϕ*_1_, *ϕ*_2_, *χ*) space. No phase separation occurs in the 2 *> χ >* −8 range. Central column: cross sections of phase space at *χ* = 12 (top) and *χ* = −10 (bottom) corresponding to phase diagrams with critical points (hollow circles), spinodal (dark blue region), binodal (light blue region), and tie lines (black dashed lines) plotted. In the segregative case, the individual solutes demix into separate compartments, while in the associative case, they colocalize in condensates. Right column: lattice model illustration of the phase-separation configurations.

In a system with two solutes that phase separate into two compartments, a definition of tie-line gradient is natural. Denote dilute and dense phase concentrations of the two solutes as $\left(\;\phi_{1}^{\text{dilute}\;},\phi_{2}^{\text{dilute}\;}\;\right)$ and $(\;\phi_{1}^{\text{dense}\;},\phi_{2}^{\text{dense}\;}\;)\;$ the gradient of the tie line is simply $k\equiv \left(\phi_{2}^{\text{dense}\;}-\phi_{2}^{\text{dilute}\;}/\phi_{1}^{\text{dense}\;}-\phi_{1}^{\text{dilute}\;}\right)$ since it connects both points. In the case where both solutes have low dilute phase concentrations, we approximately have $k\approx \left(\phi_{2}^{\text{dense}\;}/\phi_{1}^{\text{dense}\;}\right)$, i.e., the volume ratio of solutes in the dense phase. We use a minimal model (Appendix A 1) to illustrate the phase-space structure of the Flory-Huggins system. Setting solutes to unit length *N*_1_ =*N*_2_ =1, and assuming inert solvent *χ*_01_ = *χ*_*02*_, the only relevant parameter is *χ*_12_ ≡ *χ*. We calculate the spinodal region analytically using the Hessian and compute the binodal and tie lines numerically via convexification of *f (ϕ*_1_, *ϕ*_2_) [17]. These two boundaries intersect and are parallel to one another at critical points. When plotting tie lines in the phase diagram, it is worth noting that they can appear curved when a logarithmic concentration scale is used and are straight only in linear units. The complete phase space of the minimal model is characterized by an associative branch (where both solutes are enriched in one phase) at χ < −8 with positive tie-line gradients and a segregative branch (where solutes are enriched in separate phases) at *χ >* 2 with negative tie-line gradients (Fig. 1). It is important to note that their phase boundaries in the dilute regime bear great semblance to each other, so measurements of phase diagram shapes in small portions of phase space cannot determine whether associative or segregative interactions are drivers of phase separation.

To derive an approximate expression for the tie-line gradient *k*, we use the full Flory-Huggins expression and diagonalize the Hessian of the free energy. We approximate *k* as the gradient of the eigenvector with the smaller eigenvalue, which corresponds to the locally preferred direction of phase separation (Appendix A 2). We then show that the sign of *k*, sgn(*k*), is sgn (*k*) = −sgn (1 + 2*χ*^Δ^) with *χ*^Δ^ ≡ (*χ*_*12*_ − *χ*_01_ − *χ*_02_/2). Assuming a dilute *ϕ*_1_ ≪ *ϕ*_2_ ≪ 1, we further obtain

(2) $$k\approx -\frac{1}{\left(1+2\chi^{\text{Δ}}\right)N_{1}\phi_{1}}.$$

To visualize the physical interpretation of *χ*^Δ^, we recall the definition of the original *χ*_*µν*_ = *(z/2)(2 ϵ_µν_* *− ϵ*_*µµ*_ *−* *ϵ*_*νν*_ */k_B_T)* with *ϵ*_*μν*_ the bare contact energy between *μ* and *ν* particles and *z* a coordination constant. Direct substitution gives *χ*Δ= (*χ*_*12 −*_ *χ*_01_ *− χ*_02_/2) = (*z*/2)[(*ϵ*_12_ + *ϵ*_00_) − (*ϵ*_01_ + *ϵ*_00_)/*k*_*B*_*T*].

**Fig. 2:**
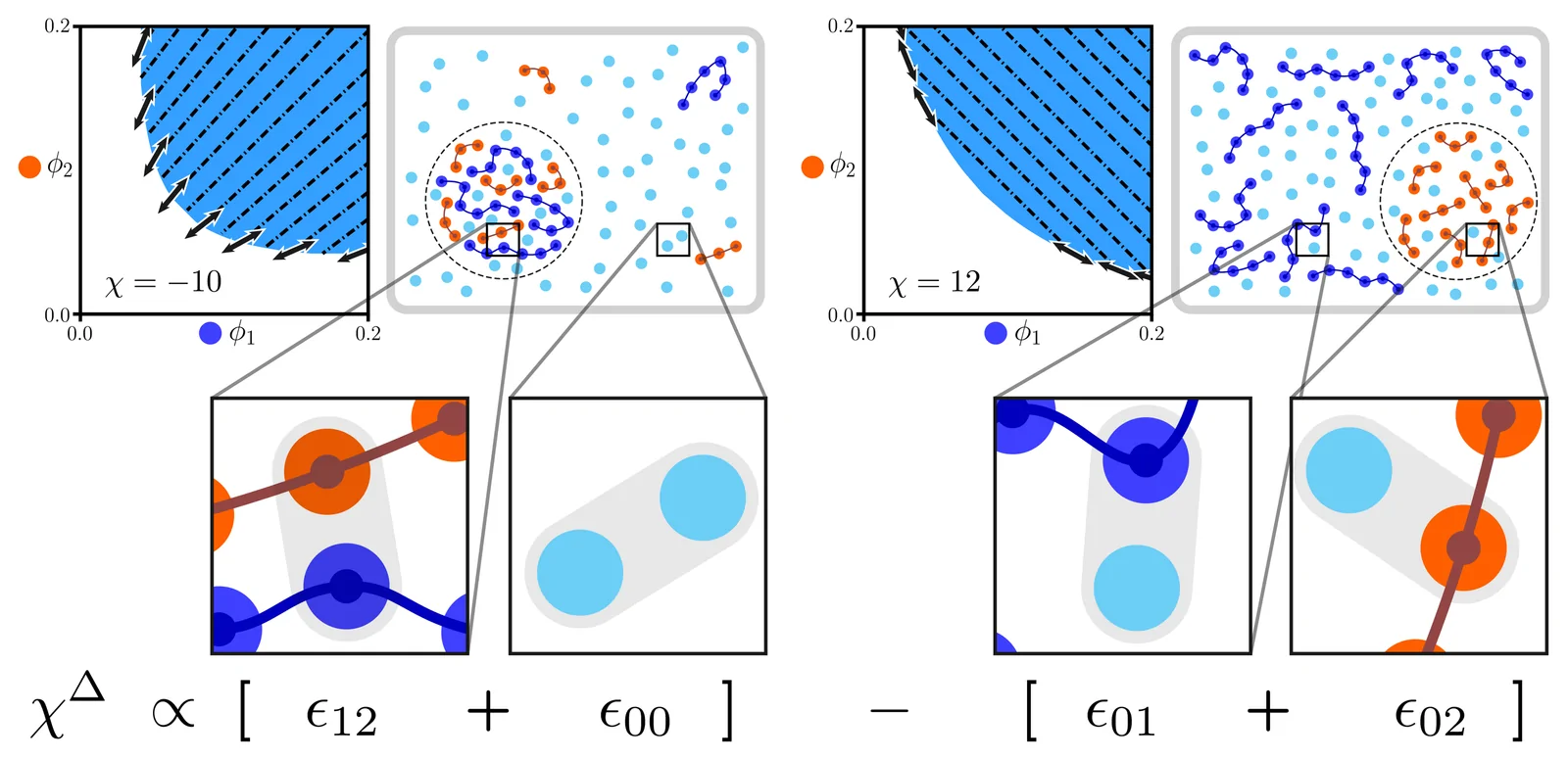
Sign of the tie-line gradient is determined by the cross-interaction energy *χ*^Δ^. Top: comparison between tie lines generated from numerical convexification (black dashed lines) and from diagonalizing the Hessian (black thick arrows), with graphical illustrations of partitioning of solutes and solvent. Dark blue regions are the binodal. Bottom: graphical illustration of the physical interpretation of the *χ*^Δ^ parameter. *χ*^Δ^ is the energy difference between forming (left) associative phases and (right) segregative phases. Notice homotypic interactions *ϵ*_11_ and *ϵ*_22_ do not enter *χ*^Δ^, although they still affect the magnitude of the tie-line gradient.

It is evident that *χ*^Δ^ signifies the energy difference between forming associative and segregative phases. We compare binodal tie lines and local Hessian eigenvectors in the dilute regime of the phase diagram (Fig. 2) and note the two agree with each other relatively well. It is important to note, however, that qualitative differences between the two exist: The tie-line gradient arises from a global equilibrium of chemical potentials and osmotic pressures of dilute and dense phases. Deviations of the tie-line direction from the locally unstable direction thus arise from a large local curvature of the free-energy surface, and this effect is most prominent at very low solute concentrations due to the logarithmic contributions (*ϕ*_*μ*_*/N*_*μ*_) ln *ϕ*_*μ*_. This term aligns the locally unstable direction parallel to the edge of the phase space (Appendix A 3) manifested in the 1*/ϕ*_1_ dependence of *k* in the limit of very small *ϕ*_1_ in Eq. (2), and as such, Eq. (2) represents an overestimation of the true tie-line gradient.

Using Eq. (2), we can estimate *χ*^Δ^ from measurement of *k* and obtain the effective interaction energy scale under the mean-field model. It should be noted that alternative ways of measuring the *χ* parameter exist in the field of polymer physics [38]. Prominent methods include neutron or light scattering [39–41] using the Hansen solubility parameter [42,43] or calculating group contributions [44,45]. Computationally, *χ* can also be obtained from simulations [46]. The *χ*^Δ^ calculated here is rather different in that it is a linear combination of individual *χ*’s, which arises due to the possibility of having compartments of differing compositions.

## III Results and Discussion

### A approach

**Fig. 3:**
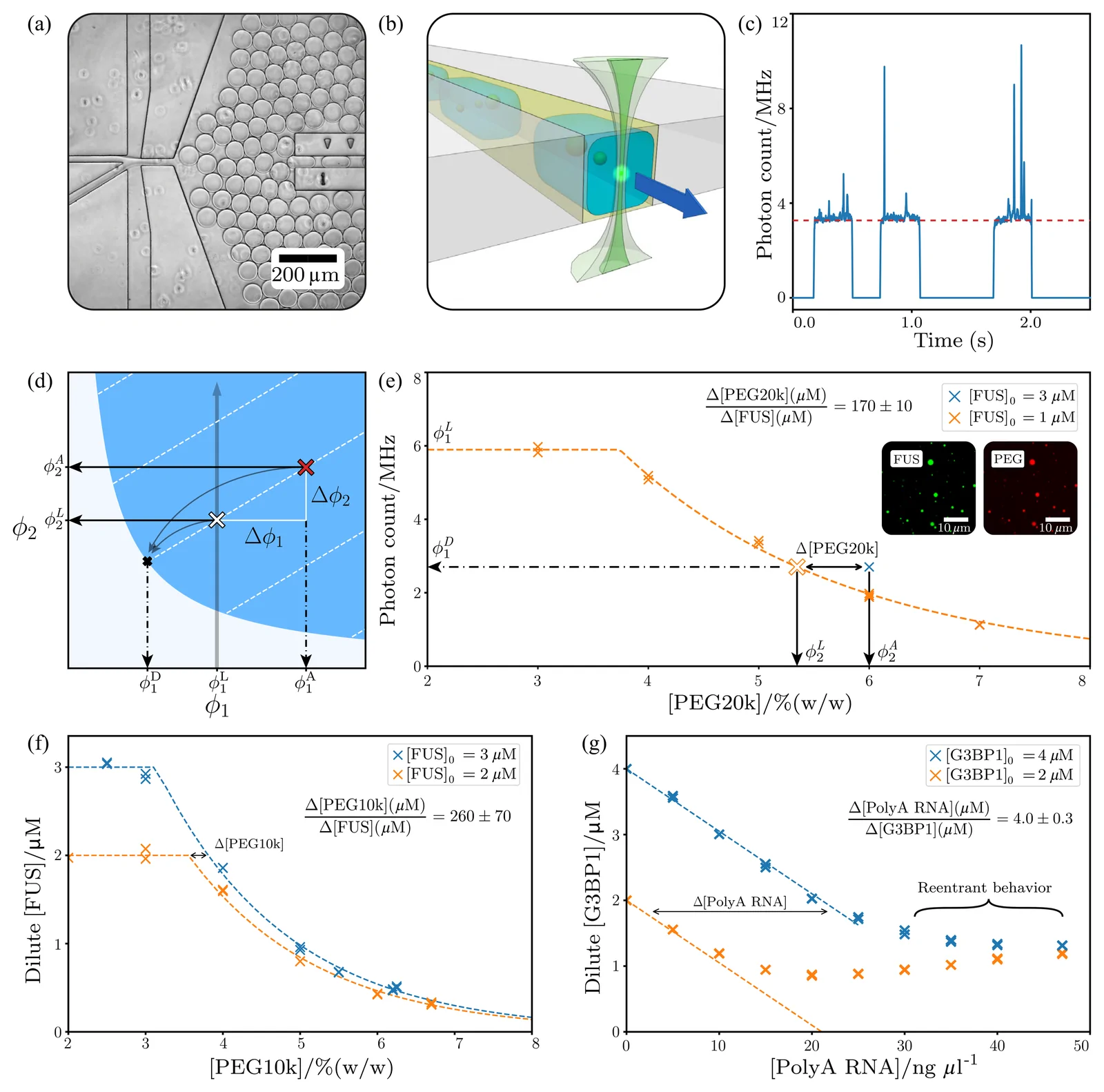
Experimental determination of tie lines through measurements of the dilute phase concentrations. (a) Bright-field-microscopy image of the microfluidic device. Droplets are generated on the left and pushed into a narrow channel on the right. Measurements are performed at the end of the channel to allow sufficient incubation time. (b) 3D illustration of the channel cross section and the confocal profile. (c) Raw signal coming from three droplets (blue solid line) characterized by plateaus from dilute phase readings and spikes from condensates passing through the confocal spot. During analysis, a 3*σ* cutoff is used to filter out the spikes to give an estimate of the dilute phase photon count averaged over approximately 100 droplets (red dashed line). Regions of low photon counts are gaps between droplets. (d) Illustration of anchored linescan. Blue region is the binodal with representative tie lines (white dashed lines). The anchor is plotted as a red cross and linescan plotted as a vertical gray solid line. By comparing the dilute phase *ϕ*_1_ concentrations (*ϕ*^*D*^), one identifies from the linescan the second point on the tie line (white cross). The gradient can then be calculated as *k* = (Δ*ϕ*_2_/Δ*ϕ*_1_). (e) Results of anchored linescan for the FUS-PEG20k system. Solid crosses are measured values and the hollow cross is interpolated. The dashed line is a phenomenological fit. Measurement errors are comparable to marker sizes. Inset: confocal microscopy images of condensates with both FUS and PEG20k labeled. (f) Serial linescans for the FUS-PEG10k system. Dashed lines are phenomenological fits. (g) Serial linescans for G3BP1-poly(A) RNA system showing an initial drop in dilute phase (G3BP1) due to phase separation and a rise at higher [poly(A) RNA] due to the reentrant effect. Dashed lines are linear low-[poly(A) RNA] fits. Legends in (e)–(g) give total starting protein concentrations in droplets.

Experimentally, we attach florescent tags to FUS and G3BP1 proteins and use a custom-built single-molecule confocal microscope platform [47] to determine the photon-count intensity from the dilute phase, which directly relates to the protein concentration; a microfluidic device is used to trap the phase-separating systems in water-in-oil droplets so that measurements can be carried out with multiple replicas [Figs. 3(a)–3(c) and Appendix B 1].

Measuring tie lines using the dilute phase concentration of one component relies on the fact that a tie line, by definition, is a line on which two points in the phase-separated space give the same dilute and dense phase compositions. As such, the problem reduces to finding two points in the phase diagram within the phase-separated region that gives the same dilute phase concentration of one of the components—say, *ϕ*_1_—assuming the phase boundary has some finite gradient. To locate two such points, we first pick a point (the “anchor”) in the phase-separated region at $\left(\phi_{1}^{A},\phi_{2}^{A}\right)$ and measure the dilute phase concentration of *ϕ*_1_ as $\phi_{1}^{D}$. We then pick a series of points (the “linescan”), keeping the total *ϕ*_1_ concentration constant at some other value $\phi_{1}^{L}$ while varying the *ϕ*_2_ concentration. We can then interpolate the linescan measurements and find the point with the same dilute phase concentration $\phi_{1}^{L}$. This pair of points allows us to construct the tie line by connecting them with gradient *k* calculated as $k=\left(\text{Δ}\phi_{2}/\text{Δ}\phi_{1}\right)=(\phi_{2}^{A}-\phi_{2}^{L}/\phi_{1}^{A}-\phi_{1}^{L})$ [Fig. 3(d)]. The anchored linescan is the minimal measurement needed to determine the tie-line gradient, and we apply this to the FUS-PEG20k system. We then extend the method by performing multiple linescans and compare dilute phase concentrations between these, this is done in the FUS-PEG10k and G3BP1-poly(A) RNA systems (Appendix B 2). Proteins are produced with an insect cell line (Appendix B 3), and a summary of molecular weights, densities, and polymer lengths of individual components used in the following calculations are listed in Appendix B 4.

### B Anchored linescan with FUS-PEG20k

We choose the anchor at $[\text{FUS}]=3\;\text{μM}\left(\phi_{1}^{A}=0.00022\right)$ and $[\text{PEG}20\text{k}]=6\text{\%}(\text{w}/\text{w})\left(\phi_{2}^{A}=0.053\right)$. The linescan is performed at $[\text{FUS}]=1\;\text{μM}\left(\phi_{1}^{L}\right)$ for [PEG20k] in the range of 3.0%–7.0%(w/w). This gives Δ [FUS] 2 μM. The linescan data are then fit to a phenomenological curve, and we extract Δ [PEG20k] =(0.65±0.05) %(w*=*w) (Appendix B 5). Thus, we obtain the tie-line gradient $k=(\text{Δ}[\text{PEG2}0\text{k}]/\text{Δ}[\text{FUS}])=(0.33\pm 0.03)\frac{\text{\%}(\text{w}/\text{w})}{\text{μM}}$ [Fig. 3(e)]. Converting to molar ratio, we get *k =*170 ± 10. Volume and mass ratios are calculated and summarized in Table I. The resulting positive tie line is surprising, since we expected PEG to act as an inert crowder [22,25], and it should thus have a higher concentration in the bulk to exert a positive net partial pressure on the condensates. We verify our results qualitatively by preparing condensates with fluorescently labeled FUS and PEG20k. Confocal microscopy (Appendix B 6) shows that condensates are both richer in FUS and PEG20k in comparison to the dilute phase, confirming our finding that FUS and PEG20k form condensates via associative interaction. Furthermore, since $\phi_{1}^{A}\ll \phi_{2}^{A}$ we use Eq. (2) to estimate *χ*^Δ^ ≈ −0.60, so FUS and PEG20k are weakly attractive, with an effective interaction of half of *k*_*B*_*T* between lattice sites.

### C Serial linescans with FUS-PEG10k

To confirm that the positive tie-line gradient is typical for PEG and not a coincidence due to the length of choice (20 kDa), we measure the tie-line gradient for FUS-PEG10k using serial linescans without an anchor [Fig. 3(f)]. Furthermore, we also do a calibration series to map intensity to actual concentrations (Appendix B 1). The tie-line gradient can be extracted (Appendix B 5) to give $k=(0.26\pm 00.7)\frac{\text{\%}(\text{w}/\text{w})}{\text{μM}}$ and molar ratio *k* = 260 ± 70, on the same order of magnitude as the PEG20k result, indicating there is a similar associative interaction between FUS and PEG10k. Using Eq. (2) at FUS 1 μM and [PEG10k] 5% (w*=*w), we obtain *χ*^Δ^ = −0.74. We further use the linescan data to reconstruct the binodal boundary and tie lines and fit the resulting phase diagram to the Flory-Huggins theory by generating numerical phase diagrams using the convex hull algorithm [17]. The fitting gives *χ*^Δ^ ≈ −0.54 (Appendix C). Moreover, we test dextran, another carbohydrate polymer, and also find an associative interaction with FUS (Appendix D).

### D Serial linescans with G3BP1-poly(A) RNA

We use single-stranded poly(A) RNA (3.0 ± 0.5 kDa) to form protein-RNA condensates with G3BP1 and carry out serial linescans [Fig. 3(g)] The low-[poly(A) RNA] region shows a steady decrease in dilute phase [G3BP1] due to phase separation, and curves up at higher [poly(A) RNA] as it enters the reentrant branch [48,49], where the additional poly(A) RNA contributes to condensate dissolution instead of formation. We perform a linear fit for the low-[poly(A) RNA] branch and the tie-line gradient is $k=(\text{Δ}[\text{poly}(\text{A})\text{RNA}]/\text{Δ}[\text{G}3\text{BP1}])=(10.5\pm 0.8)\frac{ng/\text{μl}}{\text{μM}}$ corresponding to a molar ratio of *k* 4.0 ± 0.3. This indicates a clear associative interaction between the G3BP1 and poly (A) RNA. Using Eq. (2) to extract *χ*^Δ^ leads to a contact energy of the order −70*k*_*B*_*T* at concentrations G3BP1= 4 μM and [poly (A) RNA] 20 ng*=*μl. This large value is qualitatively indicative of strong interactions. It is likely that in this regime the dense phase interaction energies are overestimated in the Flory-Huggins picture. Indeed, G3BP1 is a RNA-binding protein and once bound, there is strong spatial correlation between the molecules, and adopting a mean-field picture inevitably weakens the interactions so a larger magnitude of *χ*^Δ^ is needed to compensate for this effect.

## E Discussion

Table I summarizes the molar, volume, and mass ratios of the components in condensates as well as the estimated cross-interaction energies. For the FUS-PEG systems, the main polymeric component in condensates, by all three metrics, is PEG. This reveals that PEG does not act as a classical crowding agent and highlights a potential caveat in using PEG to induce phase separation *in vitro*: The high PEG content in condensates can affect the behavior of proteins as compared to *in vivo* condensates, so functional conclusions derived from *in vitro* experiments do not necessarily translate into *in vivo* results. The small cross-interaction energies in FUS-PEG systems indicate condensate formation is driven by weak, nonspecific attractions. For the G3BP1-poly(A) system, we find that on average, four poly(A) molecules are present for every G3BP1 molecule. However, because poly(A) has a much lower molecular weight than G3BP1, the majority of polymers in the condensate by mass and volume is instead G3BP1. The G3BP1-poly(A) system has strong, specific attractions between polymers, rendering Flory-Huggins theory inapplicable in this scenario. Comprehensive analysis of the tie-line gradient will thus require more detailed frameworks that go beyond the Flory-Huggins mean-field model. Existing examples include the Voorn-Overbeek model [50], the sticker-and-spacer model [6,51], transfer-matrix theory [52,53], and random phase approximation [54,55]. Numerical simulations could also provide more insight into these multicomponent systems [15,37,55]. The current theories, however, do not deal with tie-line gradients directly due to a lack of data in the past, and the current work will hopefully pave the way for quantitative comparison of theories and experiments through tie lines.

We further note that to give a quick assessment of the sign of the tie-line gradient, only two point measurements are needed in principle. One can first prepare a phase-separated sample and measure the dilute phase concentration of one solute, and prepare another with higher total concentration of that same solute while keeping other conditions constant. If the dilute phase concentration increases after adding the solute, this indicates a positive tie line and vice versa, while a constant dilute phase concentration simply corresponds to a flat tie line. Similar linescans can be performed in this direction as well. It is also possible to extend the current method to phase-separating systems with more solutes if their dilute phase concentration can be measured, or if their partitioning across the dilute and dense phases can be assumed to be negligible and thus simply treated as a modulating agent. Formally, in a system with *n* solutes that phase separate into two compartments, tie lines are characterized by *n*-dimensional vectors, with each entry denoting the concentration difference for each solute between phases. Exact determination of tie lines relies on the measurement of dilute phase concentrations of *n* − 1 solutes, and in such a system, simply carrying out the line scans described above while focusing on two of the *n* components results in distortion of the measured tie-line gradient due to effects of the other *n* − 2 solutes, since the two-component measurement necessarily projects the higher-dimensional space into a two-dimensional plane. This approximation can be valid if partitioning of ignored components between phases is weak, meaning the two solutes of interest should be dominant components in the true *n*-dimensional tie-line vector. This can be the case if total concentrations of ignored solutes are low, such that entropic effects dominate that disfavor partitioning into different compartments, and can be applicable in modulation of phase-separating systems by small-molecule ligands [56]. It then becomes possible to investigate modulation of phase-separation propensities of biomolecules by measuring tie-line gradients in effective ternary systems with and without the modulation, and possibly gain insight into the mechanism of action of the ligands.

## IV Conclusions

We provide a theoretical framework for connecting tie lines to interaction energies in the study of biomolecular phase separation, and a simple experimental approach to measure the tie-line gradients. The latter allows for a quantitative description of stoichiometry and nature of interactions between solutes in a ternary system. Since biological condensates contain multiple species of proteins and nucleic acids, there is a clear need for quantitative biophysical characterization of the roles that different components play in the phase-separation process, and our approach provides a method for parsing the interactions apart.

As an application of the theoretical framework, we develop an experimental method using microfluidics and a custom-built single-molecule confocal microscope which allows us to monitor the dilute phase concentration of a phase-separating protein, and therefore determine tie lines by carrying out linescans across concentration series. Our findings highlight that the protein FUS forms associative interactions with PEG with a high stoichiometry of PEG present in the condensates, and therefore contradicts previous claims that PEG is an inert crowder. Additionally, we study the biomolecular system of G3BP1-poly(A) RNA and find that our measurements agree with previous results of associative interactions between G3BP1 and RNA. We further discover that the condensates tested have a G3BP1:poly(A) RNA molar ratio of 1:4. We note more detailed models exist that account for strong binding between solutes such as the sticker-and-spacer model [51], and phase diagrams with tie lines can be computed via simulation [15].

Having developed this theoretical and experimental framework for quantifying biopolymeric interactions in phase-separating systems, many more systems may be studied to gain further mechanistic insight into the driving forces behind phase separation. Once interactions between different components are further understood, one may be able to better design therapeutics to selectively disrupt or enhance phase separation of disease-relevant biomolecules with greater mechanistic insight.

## Supplementary Material

Appendix

## Figures and Tables

**Table I:** Summary of ratios of components in condensates in different units and cross-interaction energies *χ*^Δ^. PEG is the main polymeric component in FUS-PEG condensates in molar, volume, and mass ratios, while in G3BP1-poly(A) RNA condensates, poly(A) dominates in number, and G3BP1 dominates in volume and mass. *χ*^Δ^ estimates show FUS-PEG and G3BP1-poly(A) interactions are weakly and strongly attractive, respectively.

		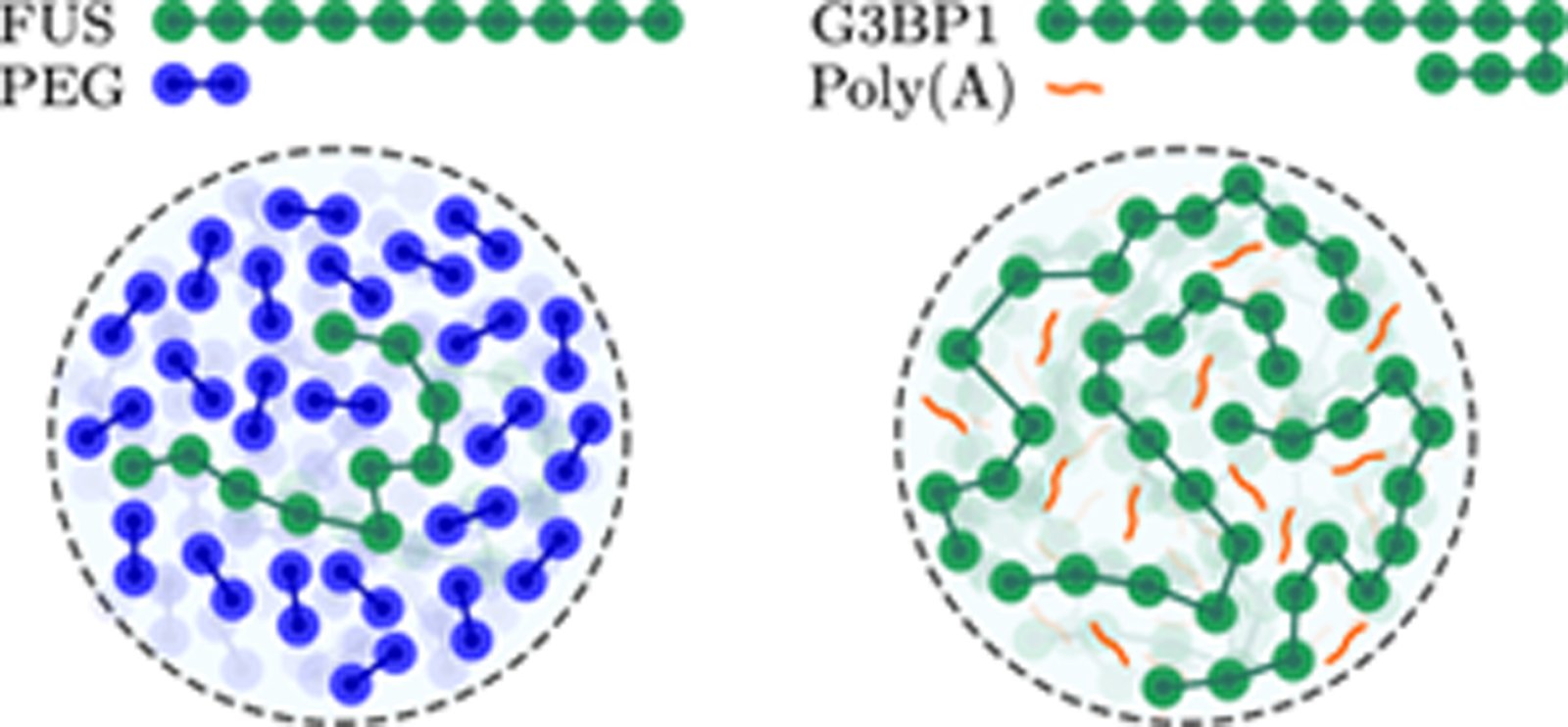			
		FUS : PEG20k	FUS : PEG10k	G3BP1 : Poly(A)	
	Molar ratio	1 : 170±10	1 : 260±70	1 : 4.0±0.3	
	Volume ratio	1 : 38±3	1 : 29±8	1 : 0.12±0.01	
	Mass ratio	1 : 32±2	1 : 24±6	1 : 0.14±0.01	
	Mean-field *χ*^Δ^	−0.60	−0.74	−70	
					
