# Supplementary material for: Tie-Line Analysis Reveals Interactions Driving Heteromolecular Condensate Formation
Source: Phys Rev X. Author manuscript; Available in PMC 2026 Sep 3. (PMC7619433; doi:10.1103/PhysRevX.12.041038)
Supplement: Supplementary file 1 [file EMS218018-supplement-Appendix.pdf]

APPENDIX A: MATHEMATICAL DETAILS

1. Minimal Flory-Huggins model

The free-energy density in the minimal model is $f(\phi_1, \phi_2) = (1 - \phi_1 - \phi_2)\ln(1 - \phi_1 - \phi_2) + \phi_1 \ln \phi_1 + \phi_2 \ln \phi_2 + \chi\phi_1\phi_2$, and ϕ_1, ϕ_2 have to fall in the physically allowed region $0 < \phi_1$, $0 < \phi_2$, and $\phi_1 + \phi_2 < 1$. We first calculate the critical values of χ, where the spinodal region is a single point, and then calculate the critical points where the spinodal and binodal meet.

The spinodal boundary satisfies the condition $\det H_{\mu\nu} = 0$ while within the physically allowed region. To relax the constraint, we instead treat the mixing energy as Gibbs free energy and use number of particles as natural variables. This gives instead

$$G(n_0, n_1, n_2) = \sum_{\mu=0}^{2} n_\mu \ln\left(\frac{n_\mu}{n_0 + n_1 + n_2}\right) + \chi \frac{n_1 n_2}{n_0 + n_1 + n_2}, \tag{A1}$$

and the critical instability condition is that any one of the three chemical potentials have a vanishing second derivative. This condition, instead of a vanishing Hessian, arises because the three components are decoupled and the Hessian of G is always 0. The constraints are simply $n_\mu \geq 0$.

In the case $\partial_1^2 G = 0$ where $\partial_\mu \equiv (\partial/\partial n_\mu)$ (or $\partial_2^2 G = 0$, these give the same expression): $(n_0 + n_1 + n_2)^2 - 2\chi n_1 n_2 = 0$. Solving for n_1 and n_2 in terms of n_0, subject to the condition that only one set of solution exists, we get $n_0 = n_0$, $n_1 = (\chi - 1)n_2 - n_0$, and $n_2 = (2n_0/\chi - 2)$. A critical interaction parameter $\chi_c^{(1)} = 2$ can be identified from the condition $n_2 > 0$. This value corresponds to the critical interaction parameter for two-component Flory-Huggins free energy. To calculate the volume fractions at $\chi_c^{(1)} = 2$, we first rewrite $\phi_\mu = n_\mu/(n_0 + n_1 + n_2)$ and then substitute $\chi = 2$. This gives

$$\begin{cases} \phi_0 = \frac{1}{2} - \frac{1}{\chi} \\ \phi_1 = \frac{1}{2} \\ \phi_2 = \frac{1}{\chi} \end{cases} \rightarrow \begin{cases} \phi_0 = 0, \\ \phi_1 = \frac{1}{2}, \\ \phi_2 = \frac{1}{2}. \end{cases} \tag{A2}$$

The concentrations thus fall onto the boundary of the physically allowed region, and this case becomes intractable if we were to use the Hessian and apply the physical constraint.

For $\partial_0^2 G = 0$, we get an equation quadratic in n_0 with discriminant $4\chi n_1 n_2[2n_1^2 + (4 + \chi)n_1 n_2 + 2n_2^2]$. Setting it to zero, we obtain another quadratic equation in n_1 with discriminant $\chi n_2^2(8 + \chi)$ from which we obtain $\chi_c^{(2)} = -8$. The concentrations at this point are

$$\begin{cases} n_0 = 2n_1 \\ n_1 = n_1 \\ n_2 = n_1 \end{cases} \rightarrow \begin{cases} \phi_0 = \frac{1}{2}, \\ \phi_1 = \frac{1}{4}, \\ \phi_2 = \frac{1}{4}. \end{cases} \tag{A3}$$

Since the second critical interaction $\chi_c^{(2)}$ has concentrations all within the physically allowed region, we can extend this to general N_1, $N_2 \neq 1$ using the Hessian. Starting with the equation $\det H_{\mu\nu} = 0$ and setting the numerator to zero, we obtain a polynomial quadratic in both ϕ_1 and ϕ_2. Taking the discriminant twice with respect to ϕ_1, ϕ_2 and setting the result to 0 gives

$$\chi_c^{(2)}(N_1, N_2) = -2\left(1 + \frac{1}{\sqrt{N_1}}\right)\left(1 + \frac{1}{\sqrt{N_2}}\right), \tag{A4}$$

and the corresponding concentrations are

$$\begin{cases} \phi_0 = 1 - \frac{1}{2(1+\sqrt{N_1})} - \frac{1}{2(1+\sqrt{N_2})}, \\ \phi_1 = \frac{1}{2(1+\sqrt{N_1})}, \\ \phi_2 = \frac{1}{2(1+\sqrt{N_2})}. \end{cases} \tag{A5}$$

It is interesting to note the functional form of $\chi_c^{(2)}(N_1, N_2)$ is very similar to that for the general two-component system:

$$\chi_c^{(1)}(N_1, N_2) = \frac{1}{2}\left(\frac{1}{\sqrt{N_1}} + \frac{1}{\sqrt{N_2}}\right)^2. \qquad \text{(A6)}$$

Next, we calculate the critical points where the spinodal and binodal meet. The interaction parameter χ has to satisfy $\chi > 2$ or $\chi < -8$, so the spinodal and binodal exist and are not single points. Since the spinodal is completely contained within the binodal region and both curves are smooth, they are cotangent to one another at critical points. The tangent of the spinodal can be worked out easily by differentiating the equation $0 = \det H_{\mu\nu} = \chi^2\phi_1\phi_2(1-\phi_1-\phi_2) + 2\chi\phi_1\phi_2 - 1$ and gives

$$\boldsymbol{T}_{\rm spi} \propto \begin{Bmatrix} \phi_1[2+\chi(1-2\phi_2-\phi_1)] \\ -\phi_2[2+\chi(1-2\phi_1-\phi_2)] \end{Bmatrix}. \qquad \text{(A7)}$$

For the tangent of the binodal, we observe that at a critical point the ends of tie lines shrink to a single point and thus must be asymptotically parallel to the direction of least stability, which is exactly the eigenvector that corresponds to the smaller of the eigenvalues of $H_{\mu\nu}$. We thus obtain

$$\boldsymbol{T}_{\rm bin} \propto \begin{bmatrix} 1-\phi_1 \\ -\phi_2 - \chi\phi_2(1-\phi_1-\phi_2) \end{bmatrix}. \qquad \text{(A8)}$$

The critical points satisfy $\boldsymbol{T}_{\rm spi} \times \boldsymbol{T}_{\rm bin} = 0$ as well as the spinodal equation (since it is on the spinodal), and the two equations have to be solved simultaneously to determine the critical point positions. Using *Mathematica*, these can be solved to give the following points, subject to the constraint that they have to lie within the physically allowed regions:

$$\boldsymbol{\phi}_c^{(1)} = \begin{pmatrix} \frac{1}{\chi} \\ \frac{1}{\chi} \end{pmatrix},$$

$$\boldsymbol{\phi}_c^{(2.\mathrm{i})} = \begin{bmatrix} \frac{4+\chi-\sqrt{(\chi+2)(\chi+8)}}{2\chi} \\ \frac{4+\chi+\sqrt{(\chi+2)(\chi+8)}}{2\chi} \end{bmatrix},$$

$$\boldsymbol{\phi}_c^{(2.\mathrm{ii})} = \begin{bmatrix} \frac{4+\chi+\sqrt{(\chi+2)(\chi+8)}}{2\chi} \\ \frac{4+\chi-\sqrt{(\chi+2)(\chi+8)}}{2\chi} \end{bmatrix}. \qquad \text{(A9)}$$

2. Tie-line direction

The Hessian is defined as $H_{\mu\nu} \equiv (\partial/\partial\phi_\mu)(\partial/\partial\phi_\nu)f$, and using the Flory-Huggins expression it is

$$H_{\mu\nu} = \begin{pmatrix} \frac{1}{N_1\phi_1} & 0 \\ 0 & \frac{1}{N_2\phi_2} \end{pmatrix} + \frac{1}{1-\phi_1-\phi_2}\begin{pmatrix} 1 & 1 \\ 1 & 1 \end{pmatrix} + 2\begin{pmatrix} -\chi_{01} & \chi^\Delta \\ \chi^\Delta & -\chi_{02} \end{pmatrix}, \qquad \text{(A10)}$$

where $\chi^\Delta \equiv (\chi_{12} - \chi_{01} - \chi_{02}/2)$. The first term is the entropic contribution from solutes, which decouples the ϕ_1 and ϕ_2 directions due to its diagonal form; the second term is the entropic contribution from the solvent and it maximally mixes the two directions; the third term has the form of an interaction matrix and its mixing or demixing tendency is a competition between the manifest self-energies $-\chi_{01}$ and $-\chi_{02}$ (in the diagonal position) and the cross-energy χ^Δ (in the off-diagonal position). In the dilute regime, we approximate $1-\phi_1-\phi_2 \approx 1$ and absorb the solute entropy into the interaction parameters, redefining the interaction matrix by writing $\begin{pmatrix} \tilde\chi_1 & \tilde\chi^\Delta \\ \tilde\chi^\Delta & \tilde\chi_2 \end{pmatrix} \equiv 2\begin{pmatrix} \frac{1}{2}-\chi_{01} & \frac{1}{2}+\chi^\Delta \\ \frac{1}{2}+\chi^\Delta & \frac{1}{2}-\chi_{02} \end{pmatrix}$. Furthermore, we absorb the segment numbers N_μ into ϕ_μ as well: $\tilde\phi_\mu \equiv N_\mu\phi_\mu$. The Hessian becomes $\begin{pmatrix} (1/\tilde\phi_1)+\tilde\chi_1 & \tilde\chi^\Delta \\ \tilde\chi^\Delta & (1/\tilde\phi_2)+\tilde\chi_2 \end{pmatrix}$. The eigensystem for the simplified $H_{\mu\nu}$ can be easily worked out, and we identify the eigenvector corresponding to the smaller eigenvalue as a proxy for the tie-line direction, since it is the direction of least free-energy increase for phase separation. The gradient of this eigenvector is

$$\frac{\Delta\phi_2}{\Delta\phi_1} = \frac{Q - \sqrt{Q^2 + 4\tilde\chi^{\Delta 2}\tilde\phi_1^2\tilde\phi_2^2}}{2\tilde\chi^\Delta\tilde\phi_1\tilde\phi_2} \qquad \text{(A11)}$$

with $Q \equiv [(\tilde\phi_1 - \tilde\phi_2) - \tilde\phi_1\tilde\phi_2(\tilde\chi_1 - \tilde\chi_2)]$. The numerator is always negative, so the sign of this gradient completely depends on $\tilde\chi^\Delta$; we thus claim $\mathrm{sgn}(k) = \mathrm{sgn}(\Delta\phi_2/\Delta\phi_1) = -\mathrm{sgn}(\tilde\chi^\Delta) = -\mathrm{sgn}(1+2\chi^\Delta)$. In experiments, we observe that the amount of proteins used corresponds to a very small volume fraction, so we make the approximation $\tilde\phi_1 \ll \tilde\phi_2$ without loss of generality. Keeping only zeroth-order terms in the numerator, we arrive at the approximate gradient

$$k \approx -\frac{1}{(1+2\chi^\Delta)N_1\phi_1}. \qquad \text{(A12)}$$

It is interesting to note that the homotypic interactions have totally disappeared. On the other hand, if one has only information on the phase boundary in the dilute regime the sign of $\tilde\chi^\Delta$ is inaccessible. To demonstrate the point, we calculate the spinodal boundary by setting the Hessian at low ϕ_1, ϕ_2 to zero, obtaining

$$\left(\frac{1}{\tilde\phi_1} + \tilde\chi_1\right)\left(\frac{1}{\tilde\phi_2} + \tilde\chi_2\right) = (\tilde\chi^\Delta)^2. \qquad \text{(A13)}$$

As such, the spinodals near the origin do not depend on the sign of $\tilde\chi^\Delta$ at all. Although phase diagrams measured in experiments are assumed to be the binodal, we assume that the binodal shape is not qualitatively different from the spinodal, so the experimentally measured dilute phase boundary alone is not sufficient to decipher the mechanisms of phase separation.

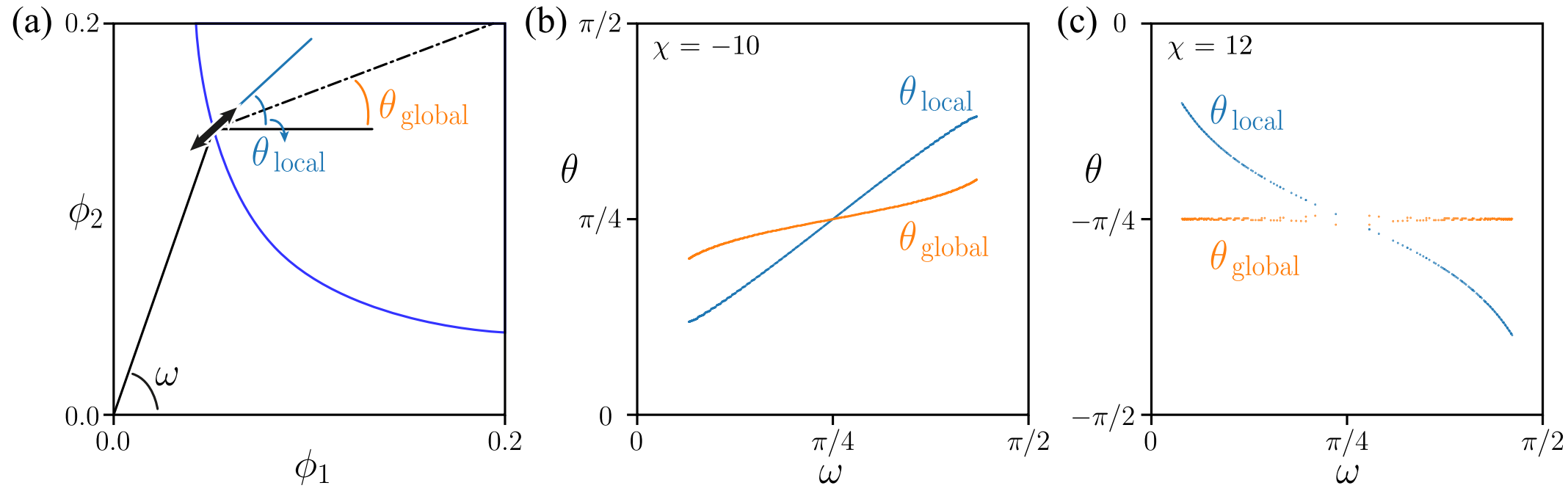

FIG. 4. Comparison between tie-line directions calculated numerically using global equilibrium conditions (orange) and analytically through the local Hessian matrix (blue). (a) Illustration of notations used. The blue curve is the binodal phase boundary, the black dot-dashed line is the numerical tie line, and the thick arrow indicates the less stable direction of the local free-energy Hessian. (b) Tie-line angles for associative phase separation with an attractive $\chi = -10$, and (c) with a repulsive $\chi = 12$. The local gradient tends to align with the $\phi_1 = 0$ and $\phi_2 = 0$ axes when the sample point is close to either axis, respectively, as a result of the high local curvature of the free-energy surface at low concentrations.

3. Comparison between numerical tie line and Hessian eigenvectors

Here we quantitatively compare tie-line directions calculated numerically from global equilibrium conditions and analytically from the local Hessian eigenvector for the minimal Flory-Huggins model. We do this for the associative case $\chi = -10$ as well as the segregative case $\chi = 12$, both of which are illustrated in the main text. We first parametrize the binodal phase boundary by the angle ω between the $\phi_2 = 0$ axis and the line connecting the origin to the point on the boundary, and next calculate the tie line and Hessian eigenvector gradients at that point as the angle between the tie line or eigenvector and the line parallel to the $\phi_2 = 0$ axis denoted as θ_{global} and θ_{local}, respectively [Fig. 4(a)]. θ_{global} and θ_{local} are plotted as functions of ω for the associative case [Fig. 4(b)] and segregative case [Fig. 4(c)]. In both cases, θ_{local} is biased toward 0 or $\pi/2$ at $\omega \approx 0$ or $\pi/2$, respectively, due to the alignment effect of the entropic contribution to the free-energy density.

APPENDIX B: METHOD AND MATERIALS

1. Home-built setup

a. Photon-counting setup

The measurement of tie lines requires a robust method for measuring dilute phase concentrations of proteins in heterogeneous mixtures of condensates and soluble monomer. In order to carry out these measurements, we utilize a droplet microfluidic technique in which we create microdroplets containing the desired concentrations of protein and copolymer and then measure these droplets on a home-built confocal setup [47]. In brief, a 488 laser line is coupled into a 60×-magnification water-immersion objective (CFI Plan Apochromat WI 60×, NA 1.2, Nikon) and emitted photons are directed onto an avalanche photodiode (APD). A motorized XYZ stage is mounted on top of the objective so that a microfluidic device may be monitored throughout the experiment. A four-inlet microfluidic device is used to generate water-in-oil droplets of varying concentrations of solutes, with one inlet connected to an oil syringe and three inlets to syringes loaded with individual solutes or a dilution buffer. After being made, droplets are squeezed into a measurement channel, and the actual measurements are made at the end of the channel to give sufficient incubation time (60–80 s) for the system to equilibrate. The confocal setup is illustrated in Fig. 5(a) and the microfluidic setup in Fig. 5(b). The signal from the APD is binned into 1-ms intervals to give out a photon intensity value in photons per sec (MHz), which is directly correlated with the concentration of protein so long as the protein is in the (1–10)-μM range where solution quenching may be neglected. The intensity trace typically has a baseline intensity (corresponding to dilute concentration of protein) and spikes corresponding to condensates passing through the confocal spot. These spikes are filtered out using a 3σ cutoff. We measure intensity profiles of approximately 100 droplets at each concentration composition, and the baseline intensity of each droplet is calculated. The intensities are binned to give an overall signal estimate [Fig. 5(c)]. To map the intensity to actual concentrations, we perform calibration series without phase separation and obtain monotonic conversion curves [Fig. 5(d)]. As far as tie-line gradients are concerned, however, this last step of concentration calibration is not necessary since two equal intensity signals directly imply equal concentrations due to the one-to-one nature of conversion curves. Furthermore, we note that our general measurement approach [Fig. 5(e)] does not depend on the exact method of dilute phase quantification and can be adapted to different setups.

b. Microfluidic device fabrication

The microfluidic device is designed using AUTOCAD software (Autodesk) and printed on acetate transparencies (Micro Lithography Services). The replica mold for

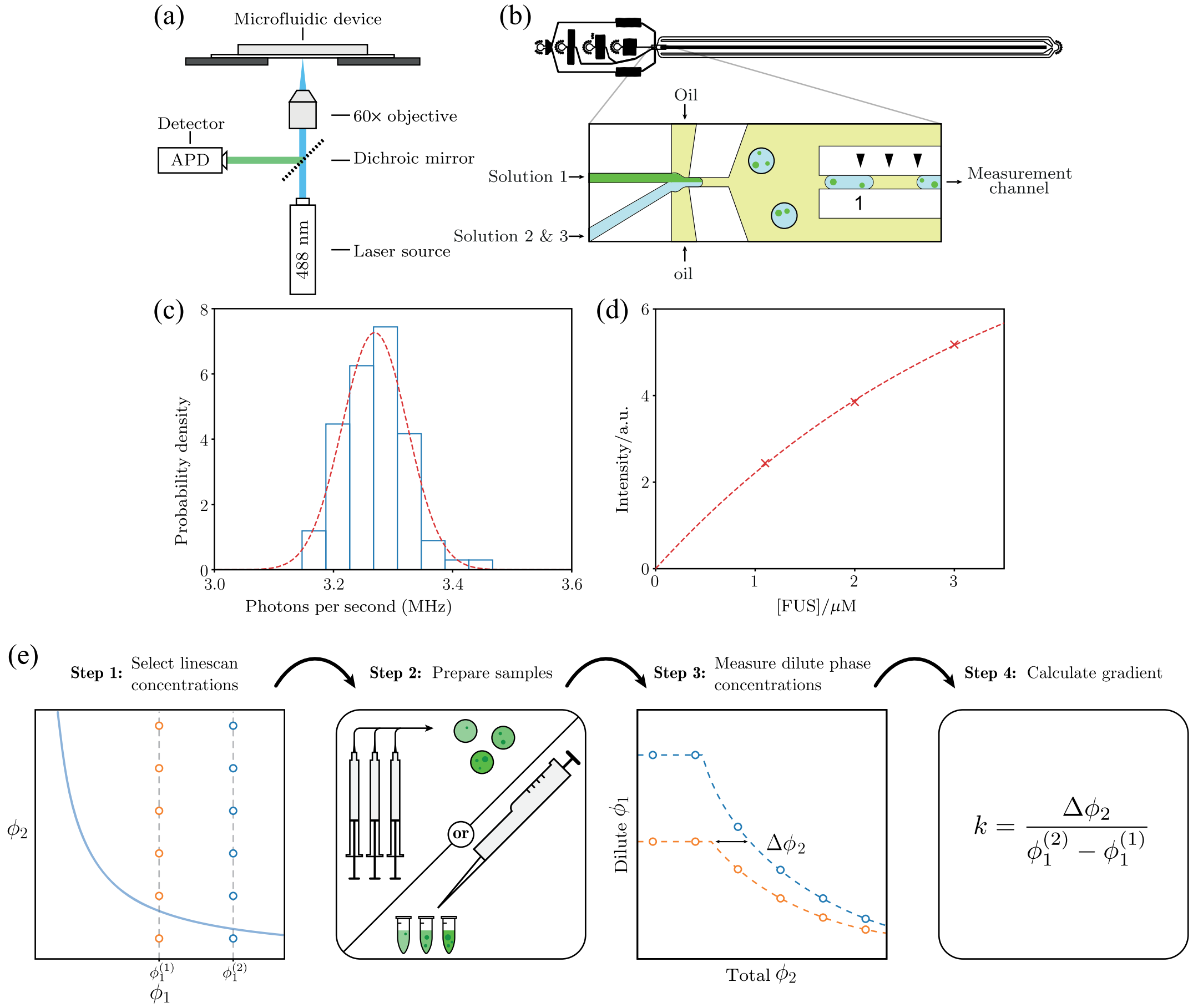

FIG. 5. Experimental overview. (a) Simplified illustration of the home-built confocal measurement setup described in detail elsewhere [47]. (b) Microfluidic device used, with an enlarged view of the droplet-generation section. (c) Histogram of mean baseline signal for individual droplets. The red dashed line is a Gaussian distribution using the mean and standard deviation calculated for the series of droplets. (d) Calibration curve for correlating signal to actual concentration. The data (red crosses) are fit to a quadratic curve (dotted line). (e) Schematic showing the general workflow of the proposed tie-line gradient measurement approach. The exact method used to quantify dilute phase concentrations is not specific to the approach.

fabricating the device is prepared through a single, standard soft-lithography step [57] by spinning SU-8 3025 photoresist (MicroChem) onto a polished silicon wafer to a height of around 25 μm. The UV exposure step is performed with a custom-built LED-based apparatus [58]. The mold is then used to generate a patterned particle desorption mass spectrometry (PDMS) slab. To this effect, the mold is cast in a 10:1 (w/w) mixture of PDMS (Dow Corning) and curing agent (Sylgard 184, Dow Corning), degassed, and baked for 1.5 h at 65 °C. The formed PDMS slab is cut and peeled off the master, and access holes for the inlet tubes are introduced using biopsy punches. The devices are then bonded to a thin glass coverslip after both the PDMS and the glass surface are activated through oxygen plasma (Diener electronic, 40% power for 30 s). After bonding, 1.5% trichloro(1H,1H,2H,2H-perfluorooctyl) silane (Sigma-Aldrich) in HFE7500 (Fluorochem) is injected into the PDMS devices to render their surface hydrophobic. After 5 min incubating in the silane solution, the solution is washed out with HFE7500, and the devices were heated at 95 °C to remove any excess oil.

2. Experimental conditions

a. FUS-PEG

The FUS proteins used in our experiments are fluorescently tagged by enhanced green fluorescent protein (eGFP). The FUS-eGFP protein is stored at 100 μM (micromolar) in 50-mM (millimolar) TRIS-HCl (trisaminomethane-hydrochloric acid), 1-M KCl (potassium chloride) under −80 °C.

TABLE II. Solution content of syringes used in microfluidic experiments for the FUS-PEG systems.

Solution	Main solute	Carrier buffer
FUS	10-μM FUS-eGFP	150-mM KCl
PEG	10%(w/w) PEG10k/20k	50-mM TRIS-HCl
Buffer	⋯	*p*H 7.4

TABLE III. Solution content of syringes used in microfluidic experiments for the G3BP1-poly(A) RNA system.

Solution	Main solute	Carrier buffer
G3BP1	11-μM G3BP1-mEmerald	85-mM KCl, 1-mM MgCl
Poly(A) RNA	290-ng/μl poly(A) RNA	50-mM TRIS-HCl *p*H 7.4
Buffer	···	1%(w/w) PEG20k

To run the microfluidic experiments, three separate syringes containing FUS, PEG, and buffer are prepared. Table II lists the content of each solution. A fourth syringe containing HFE-7500 oil (FluoroChem) and 1.2% PTFE(polytetrafluoroethylene)-PEG surfactant (RAN Bio) is additionally prepared. Initial protein solution concentrations are measured with NanoPhotometer (IMPLEN, GENEFLOW NP80) at 488-nm wavelength.

b. G3BP1-poly(A) RNA

The G3BP1 proteins used in our experiments are fluorescently tagged by mEmerald. Table III lists the content of the solutions used in the G3BP1-poly(A) RNA experiments. The fourth syringe contains the same HFE-7500 oil (FluoroChem) and 1.2% PTFE-PEG surfactant (RAN Bio) as before. The initial protein solution concentrations are measured with NanoPhotometer (IMPLEN, GENEFLOW NP80) at 488-nm wavelength.

3. Protein purification protocol

a. G3BP1 protein

A baculovirus expression system with Sf9 insect cells is used to express the recombinant pACEBac2 vector containing the cleavable (His [histidine] and MBP [maltose binding protein] tag) His-Emerald-G3BP1-MBP construct. Three days post-viral-infection, the cell culture is harvested and the pellet is resuspended in buffer A (50-mM Tris, 1.0-M KCl, 1-mM ethylenediamine tetra-acetic acid, *p*H 7.5) containing protease inhibitors and 0.1% 3-[(3-cholamidopropyl)dimethylammonio]-1-propanesulfonate. Using Dounce homogenizer, the cells are lysed, and the cell lysate is further clarified by centrifugation at 100 000 g. Supernatant containing the overexpressed protein is subjected to a three-step chromatography purification scheme. A Ni-advance column is used to capture the bulk of the fusion-G3BP1 protein. The G3BP1-containing fractions assessed by sodium dodecyl sulfate–polyacrylamide gel electrophoresis are pooled and applied to an amylose column (NEB) for further purification before being treated with the 3C PreScission Protease (Thermo Fisher) for the cleavage of the His and the MBP tags. Cleaved tags are further removed by applying the sample on a Superdex-200 Increase (Cytiva) size exclusion chromatography column in the storage buffer (50-mM Tris, 300-mM KCl, 1-mM DTT [dithiothreitol], *p*H 7.5 buffer). Pure fraction of Emerald-G3BP1 are pooled, concentrated, and snap frozen in liquid nitrogen and stored at −80 °C for the droplet assays.

b. FUS protein

The fluorescently tagged FUS protein used in this study is expressed and purified from an insect cell expression system following the previously published protocol [59]. The purified protein is stored in the final storage buffer, 50-mM Tris, 1-M KCl, 1-mM DTT, 5% glycerol, *p*H 7.4 at 100-μM protein concentration.

4. Physical parameters used in calculations

The densities, molecular weights, and segment numbers used in calculations for various components are listed in Table IV. To calculate N, we note that N represents the number of lattice sites a single molecule occupies, and we approximate the lattice volume as the volume of a single amino acid residue (density 1.35 g/ml and MW [molecular weight] 110 Da [dalton]).

5. Dilute phase data processing

a. Anchored linescan

The phenomenological curve we use to fit the linescan data is

$$y(x) = \begin{cases} a & \text{if } x < b, \\ ae^{c(x-b)} & \text{if } x > b \end{cases} \tag{B1}$$

with y denoting the signal intensity and x the total concentration of the agent (in our case, PEG20k). $y(x)$ has a plateau at low values of x and decays exponentially when x crosses some threshold b. Once we have this fit, we can compare it to the anchor values x_0, y_0 (which we know) and work out x_1 (which corresponds to the PEG20k concentration that gives the same intensity reading and thus same dilute phase FUS concentration) by writing $y_0 = y(x_1)$. We then have $x_1 = y^{-1}(y_0) = b + (1/c)\ln(y_0/a)$ and $\Delta[\text{PEG20k}] = x_0 - x_1$.

b. Serial linescan

To extract $\Delta[\text{PEG10k}]$, we again fit the data to Eq. (B1). For the two line scans, we fix a to the total [FUS], treat c as a

TABLE IV. Physical parameters used in calculations.

Solute	Density (g/ml)	MW (kDa)	N
FUS	1.35	107	909
G3BP1	1.35	87	791
PEG20k	1.125	20	218
PEG10k	1.125	10	109
Poly(A)	1.6	3	23

global fitting parameter, and b's fitting parameters for individual line scans. This allows us to extract Δ[PEG10k] in the following manner. Denote the fitted curves as $y_i(x)$ with parameters a_i, b_i, and c, with $i = 1, 2$. The condition that $y_1(x_1) = y_2(x_2)$ simply gives $\ln a_1 + c(x_1 - b_1) = \ln a_2 + c(x_2 - b_2)$ so $\Delta x_{12} \equiv x_1 - x_2 = b_1 - b_2 + (1/c)\ln(a_2/a_1)$, and it is essential that c is the same for both curves to allow the extraction to be performed. This assumes a constant tie-line gradient.

6. Confocal microscopy

To check the direction of the tie line, we image condensates with fluorescently labeled biopolymers using a Stellaris 5 confocal microscope equipped with 63× oil-immersion objective (Leica HC PL APO 63×/1.40 OIL CS2, NA 1.4). The sample solution contains 3-μM FUS and 6%(w/w) PEG20k, of which 0.5%(w/w) is fluorescently labeled. The samples are imaged in airtight imaging wells made from polydimethylsiloxane (PDMS) (Sylgard 184 kit; Dow Corning) in between (24 × 60)-mm no. 1.5 cover glass slides on the bottom (DWK Life Sciences) and (18 × 18)-mm glass slides on top (Academy). Both glass and PDMS are treated with PEG(5000)-silane (Sigma-Aldrich) by submerging them in a solution of 5 mg of PEG (5000)-silane in 20 μl of 50% acetic acid and 1 mL of ethanol for 1 h at 65 °C. The wells and glass are washed rigorously before use with water in a sonication bath.

APPENDIX C: NUMERICAL FITTING

Using the FUS-PEG10k data, we reconstruct the phase diagram and tie lines, and use the Flory-Huggins free energy to fit the interaction parameters. The results are shown in Fig. 6. The fitted interaction parameters are $\chi_{\text{FUS-water}} = 0.530$, $\chi_{\text{PEG-water}} = 0.602$, and $\chi_{\text{FUS-PEG}} = -0.063$, giving $\chi^{\Delta} = (-0.063 - 0.53 - 0.602/2) = -0.535$. This is not far from the $\chi^{\Delta} = -0.74$ calculated using the Hessian.

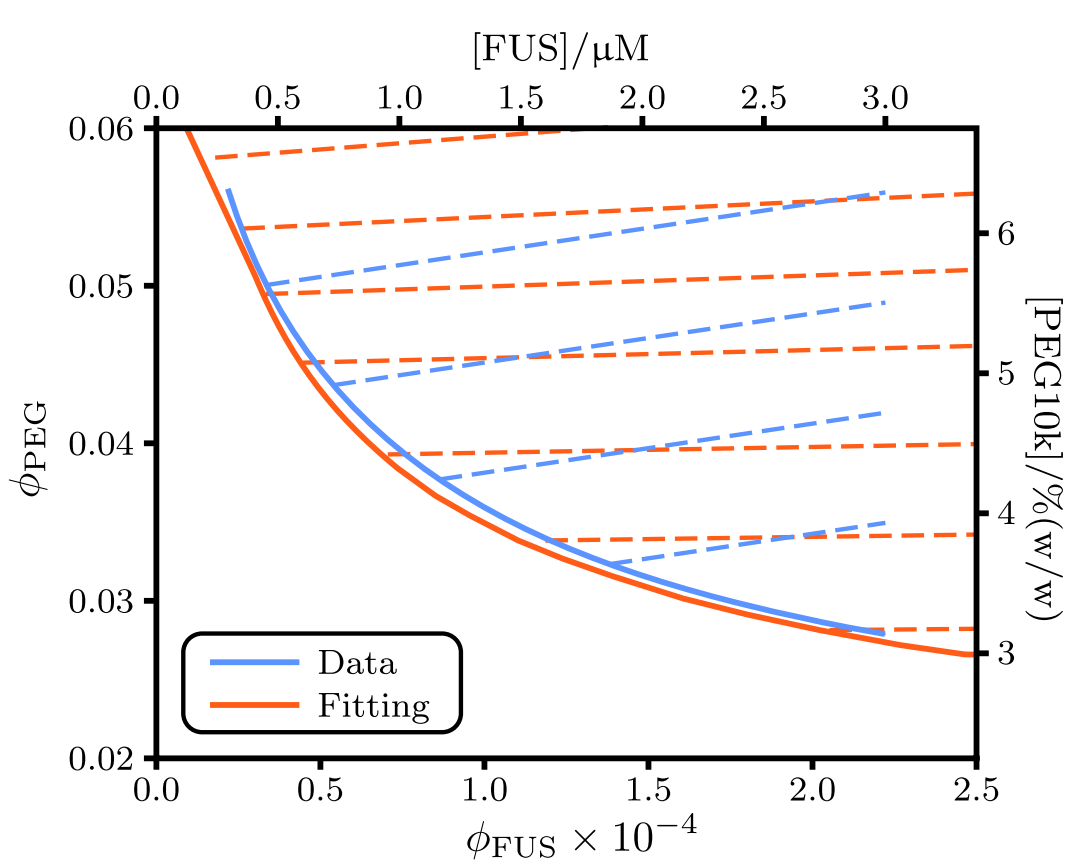

FIG. 6. Numerical fitting results of the phase boundary and tie lines.

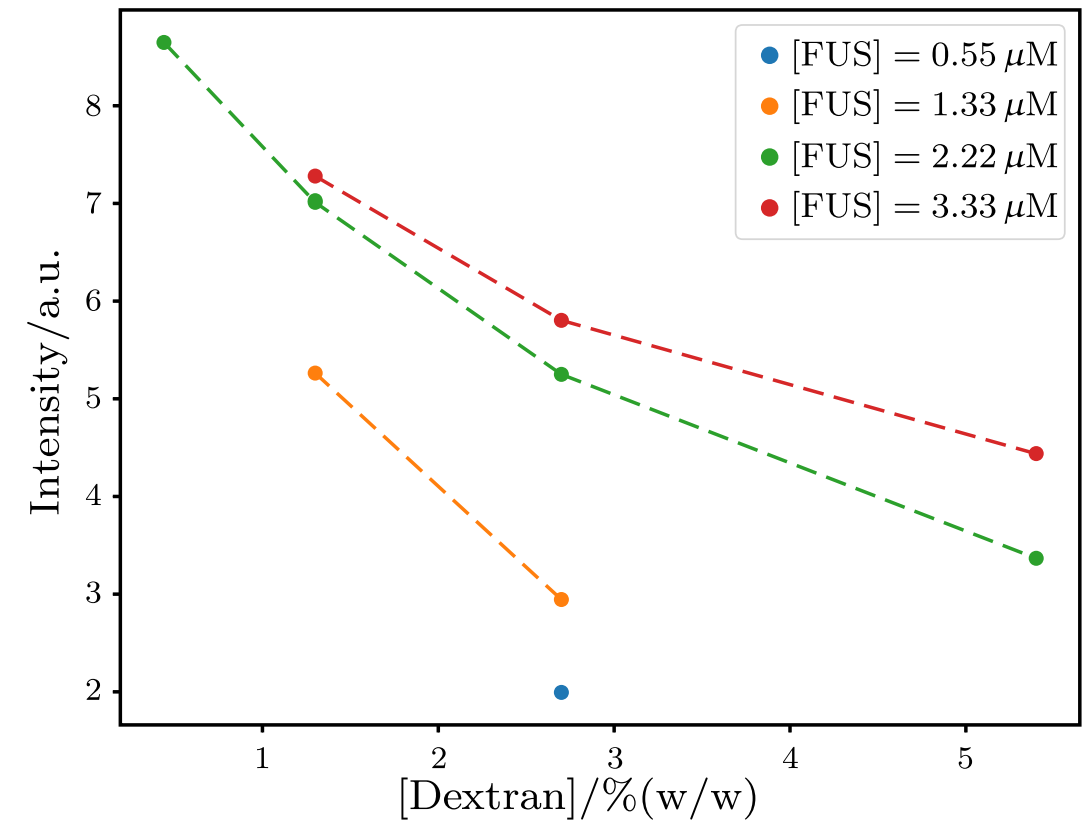

FIG. 7. Full linescan data for the FUS-dextran system.

APPENDIX D: FUS-DEXTRAN RESULTS

In addition to PEG, dextran (500 kDa) is often used to induce phase separation of FUS [60]. To test whether dextran is an inert crowder or indeed an associative polymer, we carry out linescan experiments on FUS vs dextran. The sample conditions are the same as those used in FUS-PEG systems. Our findings show that as FUS concentration is increased, the dilute concentrations are positively shifted at the same dextran concentration and that the dilute concentrations are similarly decreasing as dextran is increased for a single FUS concentration (Fig. 7). This is the same behavior observed for PEG10K and 20K, and thus concludes that dextran behaves as an associative polymer during its role in FUS phase separation.
